# Determination of 16 European Priority Polycyclic Aromatic Hydrocarbons in Doner Kebab Varieties Cooked Under Different Heating Sources

**DOI:** 10.3390/foods13233725

**Published:** 2024-11-21

**Authors:** Esra Akkaya, Hilal Colak, Hamparsun Hampikyan, Burcu Cakmak Sancar, Meryem Akhan, Ayse Seray Engin, Omer Cetin, Enver Baris Bingol

**Affiliations:** 1 Department of Food Hygiene and Technology, Faculty of Veterinary Medicine, İstanbul University-Cerrahpaşa, 34500 Istanbul, Türkiye; hcolak@iuc.edu.tr (H.C.); bingolb@iuc.edu.tr (E.B.B.); 2 Department of Gastronomy and Culinary Arts, Faculty of Fine Arts, Istanbul Beykent University, 34500 Istanbul, Türkiye; hamparsun@beykent.edu.tr; 3 Department of Nutrition and Dietetics, Faculty of Health Sciences, Istanbul Esenyurt University, 34510 Istanbul, Türkiye; burcucakmak@esenyurt.edu.tr (B.C.S.); meryemakhan@esenyurt.edu.tr (M.A.); 4 Department of Gastronomy and Culinary Arts, Faculty of Fine Arts, Istanbul Gelisim University, 34310 Istanbul, Türkiye; ascetin@gelisim.edu.tr; 5 Department of Nutrition and Dietetics, Faculty of Health Sciences, Istanbul Rumeli University, 34570 Istanbul, Türkiye; omer.cetin@rumeli.edu.tr

**Keywords:** polycyclic aromatic hydrocarbons, doner kebab, GC-MS, benzo[a]pyrene, PAH4, heating source

## Abstract

Doner kebab is a traditional Turkish meat product produced from lamb, beef or poultry meat seasoned with a blend of spices such as salt, black pepper, cumin, thyme and/or sauces. The aim of this study was to determine 16 EU priority polycyclic aromatic hydrocarbons (PAHs) in doner kebabs cooked under four different heating sources (electricity, open gas, wood and charcoal grilling). For this purpose, 200 meat doner and 200 chicken doner kebab samples were obtained randomly from various buffets and restaurants located in Istanbul and analyzed by means of GC-MS. According to the results, benzo[a]pyrene and PAH4 levels, which are important PAH compounds as biomarkers, were significantly higher in chicken doner than in meat doner (*p* < 0.05). The highest occurrence of benzo[a]pyrene and PAH4 in meat and chicken doner samples was in the charcoal heating source, whereas the lowest occurrence was detected in electric grilling. In terms of all PAH compounds, cooking with an electric heating source caused the formation of fewer PAH compounds in doner kebab samples. Consequently, the fat content of fatty meat products such as doner kebab should be reduced, the contact of fat with the heating source (especially flame) and dripping of fat to the source should be prevented and overcooking of meat should be avoided.

## 1. Introduction

Doner kebab is a traditional Turkish and Middle East meat product, widely consumed in many parts of the world and produced from lamb, beef or poultry meat seasoned with a blend of spices such as salt, pepper, cumin, thyme and/or sauces. Whole meat (1–6 mm thickness) or ground meat and a certain amount of tallow, blended with seasoning ingredients, are impaled on a vertical doner sticks and formed into a cylindrical shape [1,2,3]. The raw doner block is positioned vertically in front of a heating source such as open gas, electricity or charcoal and slowly rotated to grill the surface. The cooked surface of doner is shaved off into thin slices and served either on a plate with rice, chips, pickle and salad or in bread with sliced tomatoes, onions, chips and lettuce [4,5].

During the cooking of doner kebabs (or any grilled, roasted or barbecued meat), some harmful compounds such as polycyclic aromatic hydrocarbons (PAHs) may be formed due to the incomplete combustion and thermal decomposition of organic matter [6,7,8,9]. Out of hundreds of PAHs, 16 PAH compounds have been considered to be priority because of their toxicological and carcinogenic properties, defined by the US Environmental Protection Agency (EPA) as EPA 16 PAH and by the European Food Safety Authority (EFSA) as EU 15 + 1 PAH [10]. The EU 15 + 1 PAHs are as follows: benzo[a]pyrene (BaP), benzo[a]anthracene (BaA), benzo[b]fluoranthene (BbF), chrysene (CHR), benzo[k]fluoranthene (BkF), dibenzo[a,h]anthracene (DhA), indeno[1,2,3-cd]pyrene (IcP), benzo[g,h,i]perylene (BgP), benzo[j]fluoranthene (BjF), cyclopenta[c,d]pyrene (CPP), dibenzo[a,l]pyrene (DlP), dibenzo[a,e]pyrene (DeP), dibenzo[a,i]pyrene (DiP), dibenzo[a,h]pyrene (DhP), benzo[c]fluorene (BcL) and 5-methylchrysene (5MC) [11].

In 2005, the European Union (EU) announced BaP as a marker of the carcinogenic PAHs in particular foods (oils and fats, sea foods, heat-treated meat and meat products, etc.) with maximum limits [12]. In 2008, it was declared in EFSA CONTAM Panel that the occurrence of BaP alone was not a suitable indicator for all the genotoxic and carcinogenic PAHs in food [13]. Thus, in addition to BaP, three other PAHs (BaA, BaF and CHR) were admitted as a new marker named as PAH4 by EU regulation in 2011, and now the relevant regulation comprises both the BaP and PAH4 maximum limits in certain foods [14]. The International Agency for Research on Cancer (IARC) classified BaP, a PAH4 compound, as carcinogenic to humans (IARC Group 1), while other PAH4 compounds—BaA, BbF and CHR were probably carcinogenic (IARC Group 2A). Moreover, EFSA [13], indicating PAH8 (BaP, BaA, BaF, CHR, BkF, BgP, IcP, DhA) as a marker of PAHs in food, emphasized that PAH4 levels provide sufficient accuracy for the assessment [10,15].

PAH compounds commonly occur in foods as a consequence of thermal processes used in the preparation and production of food. Processes such as grilling and barbecuing, smoking, frying, roasting, baking, toasting and drying applied to food can be considered as important sources of PAH contamination in addition to environmental sources. The formation of PAHs in foods is influenced by a number of factors such as food components, methods used for the preparation of food (grilling, frying, smoking, roasting, etc.), temperature and time of cooking, type of heating source (gas, electricity, wood, charcoal), distance from the heating source and drainage of fat [11,16,17,18,19,20].

Among these factors, the type of heating source with different cooking methods has a critical influence on the formation of PAHs in food, as it affects both the temperature and the degree of incomplete combustion that occurs during cooking. Direct contact of food, especially fatty meat and meat products, with flame leads to the accumulation of PAH compounds on the surface of the meat due to pyrolysis of the fats in the meat at high temperatures. Furthermore, PAHs from fat dripping onto the flame or coals are carried back on the meat. Moreover, the surface area in contact with the flame is another important factor affecting PAH formation [8,9,18,21,22,23,24,25].

In terms of heating source, cooking on charcoal or wood produces the highest levels of PAHs due to smoke and direct exposure to flame. In gas and electric sources, where lower PAH levels are detected, relatively more PAH formation is observed in gas cooking due to fat evaporation and incomplete combustion. Electric and infrared cooking methods are the best at minimizing PAH formation due to the absence of smoke and combustion, but PAHs can still occur from fat evaporation and high temperatures. In addition, the vertical rotisserie technique used for cooking meat products such as doner kebab can provide consistent cooking and controlled temperature depending on the preferred heat source, which results in moderate levels of PAH formation. However, there is a high risk of PAHs in this setup due to fat dripping and overcooking [10,25,26,27].

In the light of these data, the aim of this comprehensive study in Türkiye was to determine 16 EU priority PAH compounds’ occurrence in meat doner and chicken doner kebabs cooked under different heating sources (electricity, open gas, wood and charcoal grilling) by means of GC-MS. 

## 2. Materials and Methods

### 2.1. Sampling

In this study, a total of 400 doner kebab samples (200 meat doner and 200 chicken doner), including 50 open gas, 50 electric, 50 wood and 50 charcoal grill samples for each of meat and chicken doner, were randomly obtained from various buffets and restaurants located in Istanbul Türkiye. All samples (approximately 250 g for each) were selected from well-done (unburned/not over grilled), 3–5 min grilled doner samples cooked approximately 12 ± 2 cm away from the heating sources. After the sampling process, the doner samples were placed in polyethylene vacuum bags, vacuumed and held at −18 °C in the dark until the analysis day.

### 2.2. Determination of PAHs

#### 2.2.1. Chemicals and Standards

All solvents (picograde quality), poly(acrylic acid), sodium salt-graft-poly(ethylene oxide) as a drying material and silica gel were obtained from Sigma-Aldrich (St. Louis, MO, USA). A standard mixture (500 µg/mL) of isotope-labeled (^13^C and ^2^H) PAH compounds (BaA, BaP, CPP, BbF, BkF, BjF, DhA, IcP, BgP, DlP, DeP, DiP, DhP, BcL, CHR, 5MC) was obtained from LGC standards (Wesel, Germany).

#### 2.2.2. Extraction Procedures

All analysis steps were performed according to Jira et al. [28]. The procedure for extraction and GC-MS analyses are summarized as follows. For accelerated solvent extraction (ASE), 5 g of doner samples was minced and homogenized with 5 g of the drying material poly(acrylic acid), sodium salt-graft-poly(ethylene oxide), and then transferred to 33 mL extraction cells. A 50 µL of a ^13^C-PAH standard mixture and fluorinated PAHs were used as the internal standard. The extraction procedure was conducted using Accelerated Solvent Extractor System (ASE 200, Dionex, Sunnyvale, CA, USA), with *n*-hexane under 100 bar at 100 °C for 10 min. Subsequently, the solvent was evaporated under nitrogen stream (in a 40 °C water bath). The obtained ASE extract was dissolved in 4.5 mL cyclohexane/ethylacetate (50:50 *v*/*v*) after the evaporation step and filtered through polytetrafluoroethylene filter (pore size: 1 µm) for gel permeation chromatography (GPC). The GPC column was filled with Bio-Beads S-X3 (weight of 60 g, 200–400 mesh). A flow rate of 5 mL/min was applied to elute the samples cyclohexane/ethylacetate (50:50 *v*/*v*), and the eluates were evaporated in a nitrogen stream until dryness. Then, 1 mL of cyclohexane was added to dissolve the dried GPC eluate. In order to remove more polar substances, solid phase extraction (SPE) was applied as a clean-up step. For column preparation, 1 g of the dried (550 °C, 12 h) and deactivated (with 15% water) silica was filled into 8 mL commercial SPE columns (12 mm i.d.). Afterwards, 3 mL of cyclohexane was used for conditioning the columns, and the samples were eluted with 10 mL cyclohexane.

#### 2.2.3. GC-MS Analysis

After the evaporation of cyclohexane, 1 mL of isooctane and 50 µL of the PAH standard mixture were added to the dried eluate of SPE, and this obtained sample was concentrated to a volume of 50 µL under nitrogen stream. Reagent and blank samples were analyzed simultaneously instead of real samples to determine existing PAHs in parallel to each series of samples undergoing the extraction and cleaning steps. A GC/High-Resolution Mass Spectroscopy (GC/HRMS, Agilent, Santa Clara, CA, USA) analysis of PAH was performed on an HP 5890 II gas chromatograph with a split/splitless injection port. Separation was carried out on a TR-50MS column (10 m × 0.1 mm × 0.1 µm) with an injection temperature of 320 °C and an injection volume of 1.5 µL. Helium was used as a carrier gas (constant flow of 0.6 mL/min). The identification of PAH by GC/HRMS was conducted by means of a DFS High-Resolution GC/MS (Agilent, Santa Clara, CA, USA) working in the electron impact (EI) positive ion mode using an electron energy of 45 eV. The ion source and the transfer line temperatures were maintained at 280 °C and 300 °C, respectively.

#### 2.2.4. Method Validation

Recoveries were estimated by comparing the difference between spiked and unspiked samples with the known amount of added PAHs standard. In this context, the samples were spiked with serial solutions of standards ranging from 0.2 to 5.0 µg/kg in doner samples for 30 min before extraction, whereas an unspiked doner sample was used as the control.

Average recoveries for all PAHs ranged from 85% to 98%. The relative standard deviations were in the range of 4–16%. Limits of detection and quantitation (LOD and LOQ) ranged from 0.004 to 0.01 µg/kg for BaA, BaP, CPP, BbF, BkF, BjF, BgP, BcL, CHR, 5MC and 0.02 to 0.04 µg/kg for DhA, IcP, DlP, DeP, DiP and DhP. LOD, LOQ and recovery values were acceptable according to the criteria set by the European Commission Regulation [14].

### 2.3. Fat Analysis

Fat analyses of doner samples were determined by the Soxhlet procedure according to the AOAC 991.36 method [29].

### 2.4. Statistical Analysis

The General Linear Model (GLM) procedure of SPSS 21.0 (SPSS Inc., Chicago, IL, USA) was used to determine statistical differences between groups for cooking type and meat type. In cases where interactions were significant, ANOVA (one-way analysis of variance) and Duncan test were applied to control the significance of the difference between groups.

## 3. Results and Discussion

PAHs’ formation in meat doner and chicken doner samples cooked under different heating sources was investigated by GC-MS. BaP levels were used as an indicator of carcinogenic PAH contents in foods by the EU from 2005. As it has been revealed that foods containing PAHs do not always involve BaP, both PAH4 and BaP have been used as a marker of other PAH compounds in foods by the European Commission regulation on the maximum levels for certain contaminants in food, published on 25 April 2023 (2023/915). In this context, the permissible maximum levels of BaP and PAH4 in smoked meat and meat products set by the EU regulation are 2 and 12 µg/kg, respectively [30]. 

The fat content of doner kebab is a decisive factor determining the quality of the product. Therefore, the limit of fat content values specified in the Turkish Food Codex (TFC) Communiqué on Meat, Prepared Meat Mixtures and Meat Products is stated as a maximum level of 25% and 20% for meat and chicken doner, respectively [31]. In the present study, fat contents of meat and chicken doner samples varied from 19.57 to 26.32% and from 18.68 to 22.47%, respectively. The fat analysis results of both meat and chicken doner samples collected within the scope of this study were determined as slightly higher than the limit values specified in TFC.

The PAH levels of meat and chicken doner kebab samples are shown in Table 1 and Table 2. Regardless of the meat type, higher levels of PAH compounds were formed in doner kebab samples cooked with different heating sources when grilled with wood or charcoal fire, whereas samples grilled in open gas and electric heat sources had a lower amount of PAH compounds. There was no significant difference in PAH compounds of doner kebab samples between electric and gas heat sources (*p* > 0.05). However, a significant effect was observed for only the amounts of BjF, CHR, PAH4 and total PAH compounds in samples cooked using an electric heat source. The effect of heating source on the formation of PAH components caused significant differences (*p* < 0.05), while the observed PAH levels were ordered from most to least as follows: charcoal > wood > gas > electricity (Table 1).

The effect of meat type on PAH formation was much higher in chicken doner kebab than in meat doner kebab for all heating sources (Table 1). Although the fat content in the muscle tissue of poultry meat is lower than that of beef, the use of both skinned chicken and thigh meat with high fat content in doner production has been associated with higher PAH formation in chicken doner kebabs.

There was a significant difference in the occurrence of all PAH compounds among different heating sources and meat types (*p* < 0.05), whereas there was no significant interaction between meat type and heating source (*p* > 0.05) except BjF, IcP, BgP, BcL and 5MC compounds (*p* < 0.05; Table 1).

**Figure 1:**
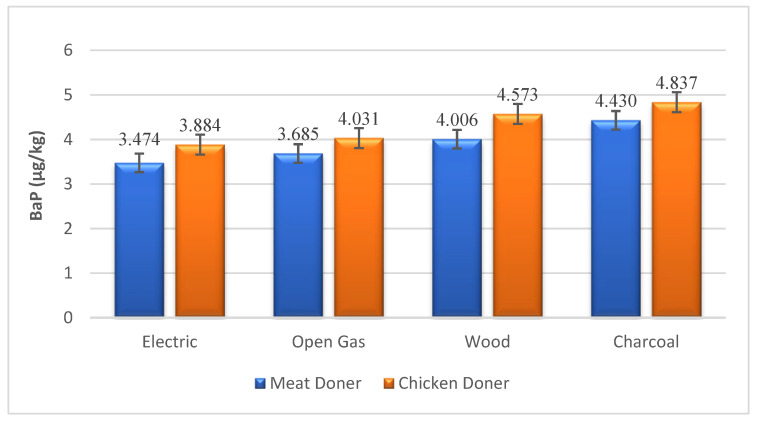
Benzo[a]pyrene (BaP) levels in doner samples under different heating sources (µg/kg).

**Figure 2:**
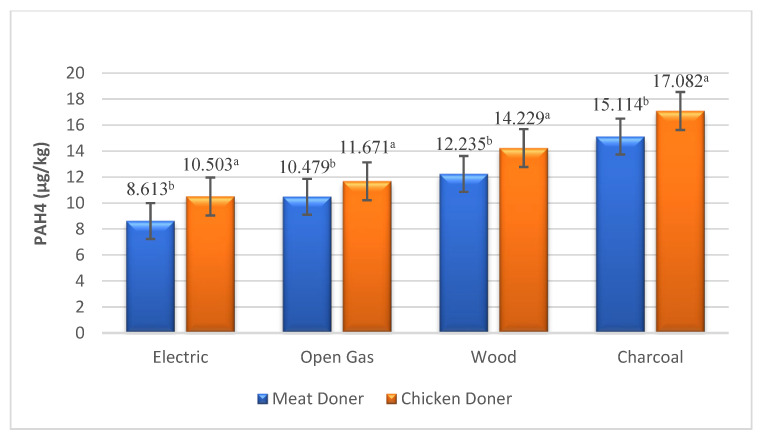
PAH4 levels in doner samples under different heating sources (µg/kg). a, b: Means with different letters are significantly different (*p* < 0.05).

**Figure 3:**
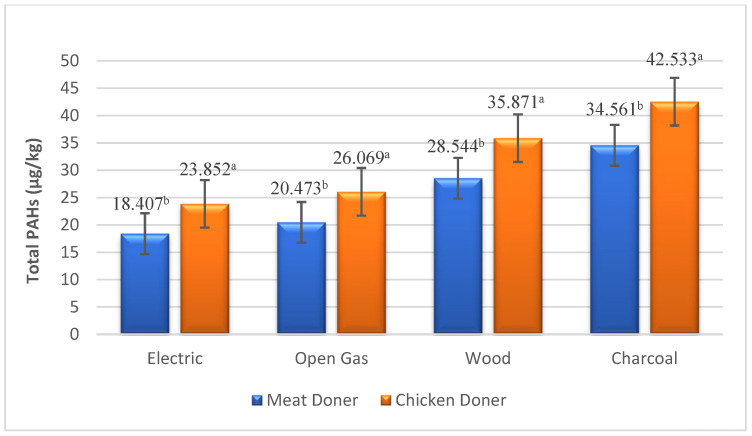
Total PAH level of doner samples under different heating sources (µg/kg). a, b: Means with different letters are significantly different (*p* < 0.05).

BaP and PAH4 levels, which are monitored biomarkers for PAH compounds, were found to be higher in chicken doner than in meat doner kebabs (Table 2). BaP and PAH4 values were determined as 3.474, 3.685, 4.006, 4.430 µg/kg and 8.613, 10.479, 12.235, 15.114 µg/kg in meat doner samples cooked in electric, gas, wood and charcoal heat sources, while detected as 3.884, 4.031, 4.573, 4.837 µg/kg and 10.503, 11.671, 14.229, 17.082 µg/kg in chicken doner samples, respectively (Figure 1 and Figure 2). A significant difference was observed for PAH4 levels between chicken and meat doner kebabs (*p* < 0.05), while no significant difference was detected for BaP (*p* > 0.05). The difference between meat and chicken doner samples in terms of total PAH levels was also determined between doner kebabs cooked under different heating sources (*p* < 0.001). Total PAH levels of doner kebab cooked in electricity, gas, wood and charcoal fire were found to be 18.407, 20.473, 28.544, 34.561 µg/kg and 23.852, 26.069, 35.871, 42.533 µg/kg in meat and chicken doner samples, respectively (Figure 3).

The minimum and maximum BaP, PAH4 and total PAH levels in meat doner and chicken doner kebab samples cooked under different heating sources are shown in Table 3. The minimum BaP levels in meat doner and chicken doner samples were 0.431 µg/kg in open gas and 0.562 µg/kg in electric grilling, while the maximum BaP levels were 14.323 µg/kg and 13.410 µg/kg in charcoal grilling, respectively. The minimum PAH4 levels in meat doner and chicken doner samples were 2.402 µg/kg and 4.841 µg/kg in electric grilling, whereas the maximum PAH4 levels were 25.311 µg/kg and 28.433 µg/kg in charcoal grilling, respectively.

In the light of these results, the highest BaP and PAH4 occurrence in meat and chicken doner samples was in the charcoal heating source, whereas the lowest occurrence was detected in the electric heating source. Studies have shown that the charcoal grilling/barbequed method used in cooking meat and meat products causes more PAH formation than other cooking methods because of pyrolysis or the incomplete combustion of organic compounds at high temperatures. The lower PAH levels detected in the doner samples can be explained by the shorter grilling time and temperature. These findings are in agreement with previous data, where PAH occurrence during charcoal grilling was reported to be dependent upon the cooking time, temperature and the fat amount [32,33,34,35,36,37].

There have been a few studies conducted on the presence of PAHs in doner kebabs in Türkiye. Terzi et al. [38] examined the effects of different cooking methods on PAH formation in doner kebabs and found that the BaP level in samples cooked in charcoal fire was 24.20 μg/kg, while this level was 5.70 μg/kg in doner samples cooked in gas flame. A total of 16 out of 40 samples were found to be above the FAO/WHO recommended maximum level of 10 μg/kg, while all samples exceeded the maximum level of 1 μg/kg that is regulated in Turkish Food Codex. They emphasized that the amount of BaP in the samples cooked in charcoal fire was much higher than the samples cooked in gas flame. In another similar study, Sahin et al. [39] analyzed each of 20 randomly collected electric-grilled meat doner and chicken doner for total PAH levels. PAH4, PAH8 and total 16PAH levels in meat and chicken doner were 2.21 μg/kg, 3.17 μg/kg, 6.08 μg/kg and 2.45 μg/kg, 3.15 μg/kg, 4.42 μg/kg, respectively. Meanwhile, BaP could not be detected in both meat and chicken doner samples. The authors associated the absence of BaP in grilled chicken samples with the lack of skin on the breast. Nevertheless, in the present study, the high level of BaP detected in chicken doner kebabs could be related to the use of both skinned chicken and thigh meat with high fat content in doner production. In agreement with our study, Karslıoğlu and Kolsarıcı [40] detected BaP in all analyzed beef doner kebab samples ranging from not detected (ND) to 7.38 μg/kg, while seven PAHs [BcFE, CPcdP, BaP, BaA, Chry, B(ghi)P, DB(a,h)A] of 16 PAHs were observed. Also, total PAH4 levels of the beef doner kebabs were in the range of 43.05–150.40 μg/kg, while total PAH8 levels were in the range of 43.05–198.10 μg/kg. They noted that the highest PAH levels were determined in the charcoal cooking method, meanwhile the lowest levels were recorded in electric cooking. It was also emphasized that the amount of PAH compounds in doner kebab varied based on fat content, cooking method and doneness degree. In another study by the same authors [41], it was reported that BaA was the PAH compound detected at the highest concentration in chicken doner samples, followed by CPcdP and B(ghi)P. The highest BaA formation was detected in chicken doner samples cooked with charcoal, while the lowest BaA level was determined in medium-done chicken doner samples cooked in an electric oven. Additionally, it was stated that BaP could not be detected in any of the heating sources. Considering PAH4 levels, medium-done chicken doner cooked in an electric oven had the lowest amount (54.50 μg/kg), while the highest level (158.70 μg/kg) was detected in well-done chicken doner cooked with a charcoal oven. In agreement with the present study, Karslıoğlu and Kolsarıcı [40,41] found that the total PAH content in chicken doner kebab was higher than meat doner kebab with the values of 302.90, 394.80 and 293.20 μg/kg in gas, charcoal and electric cooking, respectively. On the other hand, in our study, the total PAH levels of chicken doner kebabs detected with higher PAH amounts compared to meat doner were 23.852, 26.069, 35.871 and 42.533 μg/kg for electricity, gas, wood and charcoal, respectively (Table 3). This difference between the PAH levels detected in the chicken doner samples may be related to the fat content of the doner kebabs, heating source, cooking time, doneness degree and the amount of fat dripping to the heat source.

Regarding the heating source, the BaP, PAH4 and total PAH levels of the electric-grilled meat doner were in the ranges of ND-4.05 μg/kg, 2.21–86.13 μg/kg and 6.08–172.64 μg/kg, respectively [39,40], while in the present study, the mean BaP, PAH4 and total PAH levels of electric-grilled meat doner kebabs were 3.47, 8.61 and 18.41 μg/kg, respectively. However, the BaP, PAH4 and total PAH levels of meat doner grilled with gas flame were in the ranges of ND-5.70 μg/kg, 48.70–87.92 μg/kg and 82.38–196.30 μg/kg, respectively, in the studies of Terzi et al. [38] and Karslıoğlu and Kolsarıcı [40]. In the present study, the detected BaP level (3.69 μg/kg) in gas-grilled meat doner kebabs was similar with the previous studies, while the PAH4 (10.48 μg/kg) and total PAH (20.47 μg/kg) levels were lower than the findings of the conducted studies. Meanwhile, Terzi et al. [38] and Karslıoğlu and Kolsarıcı [40] emphasized that the BaP level of charcoal-grilled meat doner ranged from 4.04 μg/kg to 24.20 μg/kg depending on fat content and doneness degree. The BaP level (4.43 μg/kg) of the charcoal-grilled meat doner analyzed in this study was similar to the lowest BaP level reported by the above-mentioned researchers.

In many countries of the world, as well as in Türkiye, grilling, especially the barbecuing of meat, is a very popular cooking method. However, whether the heating source is gas or charcoal in barbecued meats shows differences in the PAH profile. The type of heating source used in barbecuing directly affects the PAH amounts and the final PAH profile of the product [15,42]. The BaP levels formed in the barbecue process using charcoal and gas were found to range from 0.76 to 7.40 μg/kg and 0.37 to 1.50 μg/kg, respectively, and the use of vertical flame-gas as the heating source resulted in the lowest concentration of PAHs [32]. This difference in PAH formation observed in gas and charcoal heating sources was attributed to the incomplete combustion of charcoal, which resulted in increased PAH levels [15]. In a similar study, Tran-Lam et al. [26] related the formation of BaP in grilled meats substantially to the grilling method. They found that the highest BaP level in meat was in those grilled directly on wood or charcoal, while the lowest BaP and total PAH contents were found in the electric stove grilling method. Accordingly, they emphasized that the use of electric stove for cooking meat may prevent the exposure of meat to strong PAH emission sources. In addition, it was stated that BaP concentrations in meat cooked with the charcoal grilling method were approximately 5–10 times higher than the values legislated in the EC [12] standard. Ali et al. [43] also reported that the chicken samples grilled on charcoal had the highest PAH4 (3.09 μg/kg) and total PAH (36 μg/kg) levels, while samples grilled on an electric heating source had the lowest mean values (0.44 µg/kg and 26.36 µg/kg for PAH4 and total PAH, respectively). Many studies have shown that replacing charcoal with gas/electricity as the heating source resulted in significant decreases in PAH concentrations [24,32,42,44]. Ahmad Kamal et al. [45] reported that the PAH4 content in gas-grilled beef samples exceeded the maximum level regulated by the EU when the temperature was 300 and 350 °C. Moreover, while BaP was detected in all gas-grilled beef samples, the level increased significantly in samples cooked at the same temperatures.

Furthermore, Sumer and Oz [46], investigating the effect of different doneness degrees (medium- and well-done) on the formation of PAHs in beef meat using direct and indirect barbecue cooking methods, reported that the highest levels of BaP (0.49 ng/g), PAH4 (6.35 ng/g) and PAH8 (11.34 ng/g) were determined in meat samples cooked well-done on the direct method of barbecuing. Moreover, the authors emphasized that an increase in the degree of doneness caused a significant increase in the PAH8 content of meat samples. Different levels of PAHs were observed in meat barbecued in both ways (directly or indirectly), and, therefore, it was recommended that care be taken during the barbecuing process. Additionally, Haiba et al. [47] detected PAHs in charcoal-grilled samples with both degrees of doneness (medium- and well-done), while grilled chicken samples had significantly higher PAH levels (9.94 ng/g) than beef (8.75 ng/g). It was also reported that beef and chicken meat samples grilled well-done had higher PAH concentrations than samples grilled medium-done. The authors generally emphasized that the degree of doneness of charcoal-grilled meats had a significant effect on increasing PAH levels.

Turkish populations generally prefer consumption of well-done meat that increases the risk of exposure to carcinogenic PAH compounds. In meats grilled for a longer period of time, especially in fatty meats, high amounts of carcinogenic PAH compounds, especially BaP, may be formed due to the effect of the high temperature of the open flame. The surface of the doner samples, which were found to contain high levels of BaP, were darker in color, that is, they had been exposed to the grill for a long time. Additionally, while collecting doner samples, it was observed that staff grilling doner kebab created a strong open flame by throwing melted fat onto the heating source. This wrong practice can obviously increase PAH formation in doner kebabs. As a matter of fact, many researchers have reported that dripping fat onto the heating source increases PAH formation [17,32,33,39,48].

## 4. Conclusions

This study evaluated the impact of cooking with different heating sources (electric, open gas, wood and charcoal grilling) on the formation of 16 priority PAH compounds in meat and chicken doner kebabs. Considering the heating sources, significantly higher levels of PAHs, especially BaP and PAH4, were detected in doner kebab samples grilled on wood or charcoal fire compared to samples grilled on open gas and electric heat sources. The higher rate of PAH formation in chicken doner kebab compared to meat doner kebab can be associated with the fat content of the collected samples, since PAHs tend to accumulate in soft tissues such as fatty tissue, skin and subcutaneous tissue. The findings indicate that the preferred heating source increases the formation of BaP and other harmful PAH compounds depending on the fat content of meat and dripping of fat into the heating source. Therefore, keeping the fat content of grilled meats low, including doner kebabs, preventing the contact of the fat with the heating source as much as possible during the grilling process and cooking the meat at lower temperatures for a longer period of time will ensure low levels of harmful PAH compounds.

## Figures and Tables

**Table 1:** Mean values and standard errors of PAHs [benzo[a]anthracene (BaA), benzo[a]pyrene (BaP), cyclopenta[c,d]pyrene (CpP), benzo[b]fluoranthene (BbF), benzo[k]fluoranthene (BkF), benzo[j]fluoranthene (BjF), dibenzo[a,h]anthracene (DhA), indeno[1,2,3-cd]pyrene (IcP), benzo[g,h,i]perylene (BgP), dibenzo[a,l]pyrene (DlP), dibenzo[a,e]pyrene (DeP), dibenzo[a,i]pyrene (DiP) and dibenzo[a,h]pyrene (DhP), benzo[c]fluorene (BcL), chrysene (CHR), 5-methylchrysene (5MC)] in doner kebab samples per studied factor (heating sources, meat type and their interaction) (µg/kg).

PAHs	Heating Source						Meat Type				Heating Source × Meat Type Interaction (*p*)
	Electric (n = 100)	OpenGas (n = 100)	Wood (n = 100)	Charcoal (n = 100)	SE	*p*	Meat Doner (n = 200)	Chicken Doner (n = 200)	SE	*p*	
**BaA**	3.400 ^c^	3.548 ^c^	4.365 ^b^	4.703 ^a^	0.114	**0.000**	3.881 ^b^	4.127 ^a^	0.081	**0.032**	0.219
**BaP**	3.679 ^b^	3.858 ^b^	4.289 ^ab^	4.634 ^a^	0.209	**0.006**	3.899 ^b^	4.331 ^a^	0.148	**0.039**	0.985
**CpP**	0.825 ^c^	0.877 ^c^	1.398 ^b^	1.651 ^a^	0.065	**0.000**	1.017 ^b^	1.358 ^a^	0.046	**0.000**	0.113
**BbF**	0.893 ^b^	1.059 ^b^	1.356 ^a^	1.527 ^a^	0.075	**0.000**	0.971 ^b^	1.446 ^a^	0.053	**0.000**	0.435
**BkF**	1.018 ^b^	1.277 ^b^	1.756 ^a^	1.938 ^a^	0.094	**0.001**	1.395 ^b^	1.600 ^a^	0.066	**0.029**	0.585
**BjF**	0.367 ^d^	0.726 ^c^	1.044 ^b^	1.512 ^a^	0.063	**0.000**	0.706 ^b^	1.119 ^a^	0.044	**0.001**	**0.001**
**DhA**	0.847 ^bc^	0.694 ^c^	1.010 ^b^	1.266 ^a^	0.065	**0.000**	0.705 ^b^	1.203 ^a^	0.046	**0.000**	0.307
**IcP**	1.038 ^c^	1.315 ^bc^	1.597 ^b^	1.993 ^a^	0.113	**0.001**	1.277 ^b^	1.695 ^a^	0.080	**0.000**	**0.010**
**BgP**	1.001 ^c^	1.056 ^c^	1.487 ^b^	1.930 ^a^	0.079	**0.000**	1.177 ^b^	1.560 ^a^	0.056	**0.000**	**0.004**
**DlP**	1.097 ^b^	0.937 ^b^	1.395 ^a^	1.510 ^a^	0.073	**0.000**	1.126 ^b^	1.344 ^a^	0.052	**0.003**	0.336
**DeP**	0.761 ^b^	0.706 ^b^	1.722 ^a^	1.878 ^a^	0.083	**0.001**	1.146 ^b^	1.387 ^a^	0.059	**0.004**	0.944
**DiP**	0.713 ^c^	0.780 ^c^	1.753 ^b^	2.048 ^a^	0.076	**0.000**	0.986 ^b^	1.661 ^a^	0.054	**0.001**	0.460
**DhP**	0.936 ^b^	0.905 ^b^	1.680 ^a^	1.807 ^a^	0.077	**0.000**	1.109 ^b^	1.554 ^a^	0.055	**0.000**	0.932
**BcL**	1.882 ^c^	1.831 ^c^	2.887 ^b^	3.261 ^a^	0.106	**0.001**	2.228 ^b^	2.702 ^a^	0.075	**0.001**	**0.006**
**CHR**	1.586 ^d^	2.609 ^c^	3.222 ^b^	5.235 ^a^	0.141	**0.000**	2.859 ^b^	3.467 ^a^	0.100	**0.000**	0.089
**5MC**	1.087 ^b^	1.092 ^b^	1.248 ^b^	1.656 ^a^	0.083	**0.001**	1.014 ^b^	1.528 ^a^	0.059	**0.001**	**0.005**
**PAH4**	9.558 ^d^	11.075 ^c^	13.232 ^b^	16.098 ^a^	0.282	**0.000**	11.610 ^b^	13.371 ^a^	0.200	**0.000**	0.713
**Total PAH**	21.130 ^d^	23.271 ^c^	32.208 ^b^	38.547 ^a^	0.440	**0.000**	25.496 ^b^	32.081 ^a^	0.311	**0.000**	0.107

SE: standard error; PAH4: sum of BaP + BaA + BbF + CHR; Means within a row with different letters (a–d) are significantly different (*p* < 0.05).

**Table 2:** The PAH levels in meat doner and chicken doner kebab samples cooked under different heating sources (µg/kg).

PAHs	Electric				Open Gas				Wood				Charcoal			
	Meat Doner (n = 50)	Chicken Doner (n = 50)	SE	*p*	Meat Doner (n = 50)	Chicken Doner (n = 50)	SE	*p*	Meat Doner (n = 50)	Chicken Doner (n = 50)	SE	*p*	Meat Doner (n = 50)	Chicken Doner (n = 50)	SE	*p*
**BaA**	3.147 ^b^	3.653 ^a^	0.102	**0.012**	3.369	3.728	0.107	0.094	4.234	4.495	0.119	0.279	4.631	4.774	0.129	0.583
**BaP**	3.474	3.884	0.195	0.294	3.685	4.031	0.195	0.376	4.006	4.573	0.205	0.167	4.430	4.837	0.238	0.396
**CpP**	0.743	0.906	0.044	0.060	0.751 ^b^	1.002 ^a^	0.053	**0.017**	1.103 ^b^	1.693 ^a^	0.079	**0.000**	1.472 ^b^	1.829 ^a^	0.085	**0.034**
**BbF**	0.742 ^b^	1.044 ^a^	0.066	**0.020**	0.852 ^b^	1.267 ^a^	0.061	**0.000**	1.072 ^b^	1.640 ^a^	0.094	**0.002**	1.220 ^b^	1.834 ^a^	0.089	**0.000**
**BkF**	0.962	1.074	0.069	0.422	1.212	1.343	0.077	0.393	1.541 ^b^	1.971 ^a^	0.110	**0.049**	1.864	2.012	0.112	0.515
**BjF**	0.243 ^b^	0.490 ^a^	0.037	**0.001**	0.660	0.792	0.051	0.194	0.844 ^b^	1.245 ^a^	0.072	**0.005**	1.077 ^b^	1.948 ^a^	0.094	**0.000**
**DhA**	0.672 ^b^	1.021 ^a^	0.050	**0.000**	0.454 ^b^	0.934 ^a^	0.066	**0.000**	0.773 ^b^	1.246 ^a^	0.086	**0.005**	0.920 ^b^	1.612 ^a^	0.072	**0.000**
**IcP**	0.843 ^b^	1.232 ^a^	0.082	**0.017**	1.250	1.380	0.099	0.516	1.070 ^b^	2.125 ^a^	0.125	**0.000**	1.943	2.043	0.148	0.736
**BgP**	0.978	1.024	0.048	0.632	0.757 ^b^	1.354 ^a^	0.073	**0.000**	1.412	1.562	0.068	0.270	1.561 ^b^	2.298 ^a^	0.121	**0.002**
**DlP**	0.882 ^b^	1.313 ^a^	0.068	**0.001**	0.904	0.971	0.062	0.594	1.320	1.470	0.090	0.407	1.398	1.622	0.073	0.123
**DeP**	0.680	0.842	0.065	0.217	0.590 ^b^	0.823 ^a^	0.055	**0.035**	1.572 ^b^	1.872 ^a^	0.090	**0.007**	1.744	2.012	0.111	0.229
**DiP**	0.449 ^b^	0.978 ^a^	0.048	**0.000**	0.490 ^b^	1.069 ^a^	0.054	**0.000**	1.363 ^b^	2.143 ^a^	0.102	**0.000**	1.642 ^b^	2.454 ^a^	0.111	**0.000**
**DhP**	0.712 ^b^	1.160 ^a^	0.065	**0.000**	0.721 ^b^	1.088 ^a^	0.049	**0.000**	1.425 ^b^	1.935 ^a^	0.084	**0.002**	1.579 ^b^	2.034 ^a^	0.109	**0.036**
**BcL**	1.745	2.020	0.081	0.087	1.270 ^b^	2.391 ^a^	0.113	**0.000**	2.793	2.980	0.126	0.463	3.105	3.416	0.113	0.171
**CHR**	1.250 ^b^	1.922 ^a^	0.089	**0.000**	2.574	2.645	0.127	0.780	2.923	3.521	0.163	0.066	4.689 ^b^	5.780 ^a^	0.184	**0.003**
**5MC**	0.886 ^b^	1.289 ^a^	0.068	**0.002**	0.934	1.250	0.090	0.080	1.094	1.401	0.088	0.080	1.142 ^b^	2.170 ^a^	0.102	**0.000**
**PAH4**	8.613 ^b^	10.503 ^a^	0.255	**0.000**	10.479 ^b^	11.671 ^a^	0.251	**0.017**	12.235 ^b^	14.229 ^a^	0.340	**0.003**	15.114 ^b^	17.082 ^a^	0.323	**0.002**
**Total PAH**	18.407 ^b^	23.852 ^a^	0.456	**0.000**	20.473 ^b^	26.069 ^a^	0.431	**0.000**	28.544 ^b^	35.871 ^a^	0.616	**0.000**	34.561 ^b^	42.533 ^a^	0.665	**0.000**

SE: standard error; PAH4: sum of BaP + BaA + BbF + CHR; Means within a row with different letters (a, b) are significantly different (*p* < 0.05).

**Table 3:** The minimum, maximum and average PAH levels in meat doner and chicken doner kebab samples cooked under different heating sources (µg/kg).

Heating Source	PAH Level (µg/kg)	Meat Doner			Chicken Doner		
		BaP	PAH4	Total PAH	BaP	PAH4	Total PAH
**Electric**	**Min**	0.620	2.402	12.212	0.562	4.841	16.141
	**Max**	9.321	15.571	26.913	8.593	15.342	34.983
	**Average**	3.474	8.613	18.407	3.884	10.503	23.852
**Open Gas**	**Min**	0.431	5.393	10.424	0.744	6.282	19.483
	**Max**	8.794	14.912	27.593	11.980	19.481	35.552
	**Average**	3.685	10.479	20.473	4.031	11.671	26.069
**Wood**	**Min**	0.691	5.422	16.990	0.792	9.804	27.452
	**Max**	10.872	21.454	39.103	11.363	26.344	49.901
	**Average**	4.006	12.235	28.544	4.573	14.229	35.871
**Charcoal**	**Min**	0.860	6.653	21.681	0.894	11.012	30.680
	**Max**	14.323	25.311	45.842	13.410	28.433	56.622
	**Average**	4.430	15.114	34.561	4.837	17.082	42.533

## Data Availability

The original contributions presented in this study are included in the article. Further inquiries can be directed to the corresponding author.
